# Regulation of Oxidative Stress Response by CosR, an Essential Response Regulator in *Campylobacter jejuni*

**DOI:** 10.1371/journal.pone.0022300

**Published:** 2011-07-19

**Authors:** Sunyoung Hwang, Minkyeong Kim, Sangryeol Ryu, Byeonghwa Jeon

**Affiliations:** 1 Department of Pathology and Microbiology, Atlantic Veterinary College, University of Prince Edward Island, Charlottetown, Canada; 2 Department of Food and Animal Biotechnology, Department of Agricultural Biotechnology, and Center for Agricultural Biomaterials, Seoul National University, Seoul, Korea; Monash University, Australia

## Abstract

CosR (*Campylobacter* oxidative stress regulator; Cj0355c) is an OmpR-type response regulator essential for the viability of *Campylobacter jejuni*, a leading foodborne pathogen causing human gastroenteritis worldwide. Despite importance, the function of CosR remains completely unknown mainly because of cell death caused by its knockout mutation. To overcome this technical limitation, in this study, antisense technology was used to investigate the regulatory function of CosR by modulating the level of CosR expression. Two-dimensional gel electrophoresis (2DGE) was performed to identify the CosR regulon either by suppressing CosR expression with antisense peptide nucleic acid (PNA) or by overexpressing CosR in *C. jejuni*. According to the results of 2DGE, CosR regulated 32 proteins involved in various cellular processes. Notably, CosR negatively regulated a few key proteins of the oxidative stress response of *C. jejuni*, such as SodB, Dps, Rrc and LuxS, whereas CosR positively controlled AhpC. Electrophoretic mobility shift assay showed that CosR directly bound to the promoter region of the oxidative stress genes. DNase I footprinting assays identified 21-bp CosR binding sequences in the *sodB* and *ahpC* promoters, suggesting CosR specifically recognizes and binds to the regulated genes. Interestingly, the level of CosR protein was significantly reduced by paraquat (a superoxide generator) but not by hydrogen peroxide. Consistent with the overall negative regulation of oxidative stress defense proteins by CosR, the CosR knockdown by antisense rendered *C. jejuni* more resistant to oxidative stress compared to the wild type. Overall, this study reveals the important role played by the essential response regulator CosR in the oxidative stress defense of *C. jejuni*.

## Introduction

*Campylobacter jejuni* is a Gram-negative foodborne pathogen belonging to the ε-subdivision of Proteobacteria [1], [2]. As a microaerophilic bacterium, *C. jejuni* is highly sensitive to atmospheric oxygen [3]. Bacterial growth in the presence of oxygen inevitably generates reactive oxygen species, such as the superoxide anion (O_2_^−^), hydrogen peroxide (H_2_O_2_) and hydroxyl radical (HO·), which can cause severe damage to biological molecules including nucleic acids and proteins [4], [5]. To detoxify reactive oxygen species, *C. jejuni* utilizes various oxidative stress defense proteins, such as superoxide dismutase (SodB), catalase (KatA), alkyl hydroperoxide reductase (AhpC) and DNA-binding protein from starved cells (Dps) [6]–[9]. While all these oxidative stress proteins play a critical role in oxidative stress defense, as the sole superoxide dismutase in *C. jejuni*, SodB is the most important enzyme in the detoxification of O_2_^−^, contributing to chicken colonization and intracellular survival of *C. jejuni* [9]–[11].

Two-component regulatory systems are one of the most important signal transduction mechanisms to modulate gene expression in bacteria [12]. The genome of *C. jejuni* NCTC 11168 contains only a small number of regulatory genes including those encoding three sigma factors, seven histidine kinases and 12 response regulators [13]. Several two-component regulatory systems that have been studied in *C. jejuni* to date are associated with bacterial motility, animal colonization and bile acid resistance [14]–[19]. Cj0355c, designated CosR (*C*ampylobacter *o*xidative *s*tress *r*egulator) in this study, is a putative response regulator essential for *C. jejuni* viability, as its knockout mutation is lethal [17], [20]. A proteomic study investigating protein expression profiles under oxidative stress showed that CosR expression is reduced after exposure to paraquat (O_2_^−^), suggesting that CosR may be involved in the oxidative stress response of *C. jejuni* [20]. Although CosR has been expected to play a critical role in sustaining the viability of *C. jejuni*, the inability to generate its knockout mutant due to lethality has limited functional characterization of this essential response regulator [17], [20].

Antisense regulation is a natural gene regulation mechanism mediated through modulation of mRNA stability, transcription, or translation in eukaryotes and prokaryotes [21], [22]. This natural process can be exploited by using synthetic antisense agents in biological research, which have significant versatility [23]–[25]. Binding of antisense agents to mRNA inhibits target gene expression by either inducing RNase H-mediated mRNA degradation or sterically interfering with ribosomal binding [23], [25]. Among antisense agents that have been developed thus far, peptide nucleic acid (PNA) is often utilized for antisense-mediated gene silencing because of its stability and high affinity for nucleic acids [23], [26], [27]. While antisense technology is frequently used in eukaryotic systems, it is seldom used in prokaryotes for gene expression control despite its usefulness. However, several recent studies have demonstrated that antisense technology can successfully control gene expression in bacteria, such as *Escherichia coli*, *Staphylococcus aureus* and *C. jejuni* [28]–[31]. As a novel approach to overcome lethality resulting from knockout mutagenesis, we performed antisense knockdown of CosR to characterize its function. Overall protein expression changes incurred by either CosR knockdown or overexpression demonstrate that CosR plays an important role in the regulation of oxidative stress response in *C. jejuni*.

## Results

### Amino acid sequence analysis of CosR and genomic organization of *cosR* flanking regions

**Figure 1:**
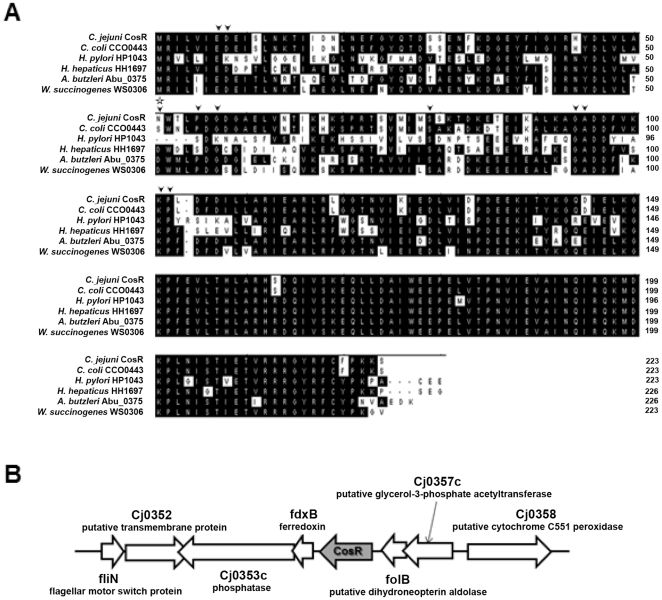
Amino acid sequence analysis of CosR homologs and genomic organization of *cosR* flanking region. (A) Multiple alignment of CosR homologs. The GenBank accession number is indicated in parentheses: *C. jejuni* CosR (Cj0355c: YP_002343793.1), *Campylobacter coli* CCO0443 (ZP_00367627.1), *Helicobacter pylori* HP1043 (AAD05966.1), *Helicobacter hepaticus* HH1697 (NP_861228.1), *Arcobacter butzleri* Abu_0375 (YP_001489319.1) and *Wolinella succinogenes* WS0306 (NP_906557.1). Based on previous studies of the HP1043 and OmpR proteins [60], [74], the highly conserved residues in the receiver domain are indicated by arrowheads. The aspartate residue which differs from the highly conserved aspartate residues in the other CosR homologs is marked with a star. (B) The analysis of *cosR* flanking region shows the lack of its cognate sensor kinase in its vicinity.

CosR is an OmpR-type response regulator essential for the viability of *C. jejuni* [17], [20]. Basic Local Alignment Search Tool (BLAST) results showed that CosR is highly conserved among *Campylobacter* species, including *C. jejuni*, *C. coli*, *C. fetus*, *C. hominis*, *C. curvus*, *C. lari* and *C. concisus* (data not shown). Interestingly, CosR homologs with high amino acid sequence similarities were found primarily in ε-proteobacteria, such as *Helicobacter* and *Wolinella* (Figure 1A). Compared with *C. jejuni* CosR, the N-terminal receiver domain of *H. pylori* HP1043 exhibited lower sequence similarity (38% identity with five gaps over 112 amino acids) than the highly conserved C-terminal DNA-binding domain (83% identity over 111 amino acids; Figure 1A). The conserved aspartate residue D8 in the CosR homologs is replaced with a lysine in HP1043, and four amino acid residues in the receiver domain (51^st^∼54^th^ amino acid residues in CosR) are deleted in HP1043 (Figure 1A). Although an asparagine residue (N51) in CosR is substituted for a conserved aspartate residue present in the CosR homologs of *Wolinella* and *Arcobacter*, other conserved aspartate residues (e.g. D8, D56, and D58) may serve as an alternative phosphorylation site (Figure 1A). Genes encoding the sensor kinase and response regulator of the two-component regulatory system are often positioned adjacent to each other; however, the potential cognate sensor kinase was not found near *cosR* (Figure 1B). In *C. jejuni*, the *cosR* gene is located downstream of ferredoxin (*fdxB*) and upstream of dihydroneopterin aldolase (*folB*; Figure 1B).

### Selective response of CosR to O_2_^−^ stress

**Figure 2:**
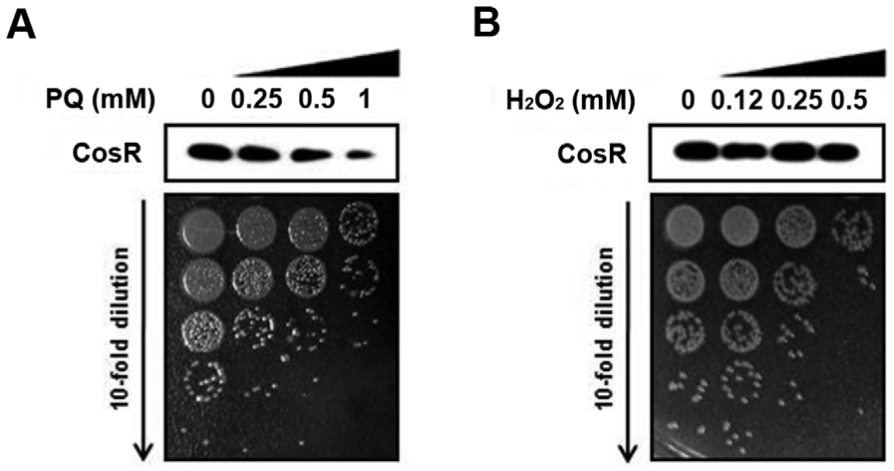
CosR expression after exposure to oxidative stress. The level of CosR protein was determined by western blotting after 1 hr exposure to paraquat (PQ; A) and hydrogen peroxide (B). The concentrations of the oxidant were indicated above the panel, and the viability changes after oxidant treatment were represented by dotting 10 µl of bacterial cultures on MH agar plates. The results are representative of three independent experiments with similar results.

According to a recent proteomic study performed by Garénaux *et al.* [20], the CosR protein level was reduced by exposure to paraquat (O_2_^−^). Consistent with the report, treatment of *C. jejuni* with paraquat significantly decreased the level of CosR protein based on western blotting (Figure 2A). To assess if CosR expression can be altered in response to peroxide stress, western blot analysis was performed after exposure to H_2_O_2_. Interestingly, H_2_O_2_ stress did not make any differences in the CosR level even at the concentrations causing similar viability changes as paraquat (Figure 2B). These findings showed that CosR expression changes responding to paraquat (O_2_^−^).

### Modulation of CosR expression with antisense PNA

**Figure 3:**
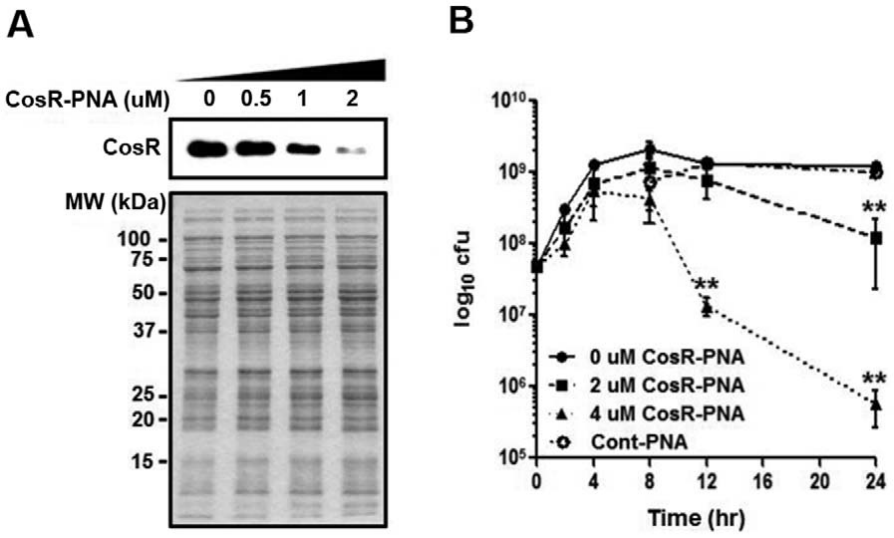
Antisense inhibition of CosR. (A) Antisense inhibition of CosR expression by CosR-PNA (upper). *C. jejuni* was incubated for 8 hrs with various concentrations of CosR-PNA. The concentration of CosR-PNA is indicated above the panel. Total protein expression at different CosR-PNA concentrations was visualized by SDS-PAGE (lower). (B) Reduction of *C. jejuni* viability by antisense inhibition of CosR. CosR-PNA (0, 2, or 4 µM) and the control PNA (Cont-PNA; 4 µM) were added at initial cultivation. After 10-fold serial dilution, aliquots were plated onto MH agar for viable cell counts. Results are expressed as the mean and standard deviation of three independent experiments. **: *P*<0.01; the statistical significance of the results was determined by two-way ANOVA analysis of variance with Bonferroni's post-tests at a 95% confidence interval using Prism software (version 5.01; GraphPad Software Inc., USA).

Due to the essentiality for *C. jejuni* viability, it has not been possible to knockout the *cosR* gene, because its knockout mutation leads to cell death [17], [20]. As an alternative in this study, antisense-mediated gene silencing was utilized to knockdown *cosR* expression. Western blot analysis confirmed that the level of CosR expression was reduced in response to the increase in the concentration of antisense CosR-specific PNA (CosR-PNA) without affecting overall protein expression based on the results of SDS-PAGE (Figure 3A). The viability of *C. jejuni* was slightly reduced with 2 µM CosR-PNA and significantly decreased with 4 µM CosR-PNA (Figure 3B). A PNA oligomer with the same number of bases as CosR-PNA, but lacking significant sequence similarity to genes in the *C. jejuni* genome, was used as a negative control. This PNA control did not reduce bacterial viability even at 4 µM (Figure 3B). These results demonstrated that antisense-mediated gene knockdown specifically controlled the level of CosR.

### Identification of the CosR regulon

**Figure 4:**
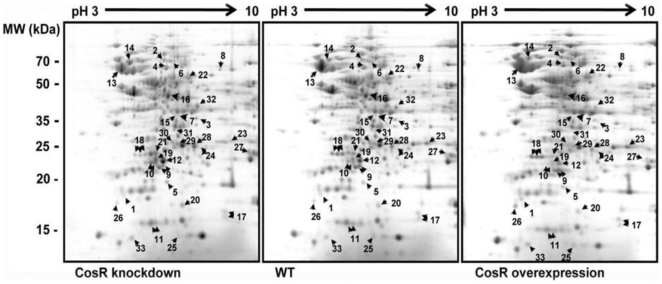
Protein expression profiles at different CosR levels. Total soluble proteins of *C. jejuni* in the CosR-knockdown, wild type, and CosR-overexpression conditions were analyzed by 2DGE. The gel is representative of three independent experiments with similar results. Spots with changes in protein level are numbered and indicated by arrows. The fold changes in protein level of each spot are described in Table 1.

**Figure 5:**
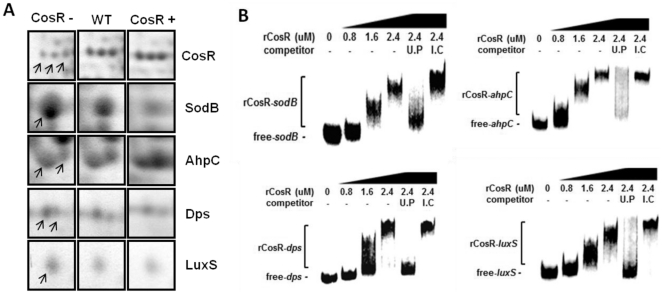
Regulation of oxidative stress defense proteins by CosR. (A) The spots of oxidative stress proteins regulated by CosR were compared at different CosR levels. The spots of CosR protein were included to demonstrate the different level of CosR in each condition. The protein spots are from 2DGE (Figure 4) and specific protein spots are indicated by arrows. “CosR−” and “CosR+” indicate CosR knockdown and CosR overexpression, respectively. (B) Binding of rCosR to the promoter region of oxidative stress genes. Radiolabeled gene probes of *sodB*, *dps*, *luxS* and *ahpC* were incubated with rCosR at different concentrations, and unlabeled DNA probes (U.P) and internal coding regions (I.C) of each target gene were used as a competitor.

To identify the CosR regulon, protein expression profiles were analyzed after CosR knockdown and CosR overexpression. The effective concentration of CosR-PNA was determined by altering the treatment time and the CosR-PNA concentration to suppress CosR expression without significant viability changes. Based on the results of western blot analysis and viability tests (Figure 3), 1.5 µM CosR-PNA was determined to be the most appropriate concentration to knockdown CosR. Alternatively, the effect of CosR overexpression was evaluated with a strain that harbors an extra copy of *cosR* in the chromosome. Quantitative real-time PCR (qRT-PCR) and western blot analysis confirmed *cosR* overexpression at the transcriptional and translational levels in the strain (Figure S1). Analysis of the protein expression profiles in the CosR-overexpression strain provided complementary evidence for the protein expression changes observed in the CosR-knockdown condition. Altered CosR levels by knockdown or overexpression influenced the expression of 32 proteins involved in various cell functions, including macromolecule biosynthesis, energy metabolism, respiration, heat shock response, regulation and oxidative stress defense (Figure 4 and Table 1). CosR appeared in the two-dimensional gel as three spots located parallel with different *p*I values (Figures 4 and 5A), presumably due to protein modifications such as phosphorylation. Based on the results of two-dimensional gel electrophoresis (2DGE), we found that the level of CosR was reduced by PNA knockdown and elevated in the CosR-overexpression strain (Figures 4 and 5A), confirming successful control of the CosR level in both CosR knockdown and overexpression conditions. CosR knockdown resulted in a 2.1-fold decrease in the protein level of PckA (phosphoenolpyruvate carboxykinase), an essential protein in *C. jejuni* [32], whereas CosR overexpression increased PckA protein levels by 3-fold (Figure 4 and Table 1). CosR affected the expression level of proteins associated with copper tolerance and gene regulation (Figure 4 and Table 1); CosR negatively regulated Cj1516, a homolog of the multicopper oxidase CueO in *E. coli*, and CprR, which is another essential response regulator in *C. jejuni* [18], [33]. Notably, CosR regulated the expression of several important proteins of oxidative stress defense in *C. jejuni*; CosR negatively regulated SodB, Dps, LuxS and Rrc, whereas AhpC was positively regulated by CosR (Figures 4, 5A and Table 1). Electrophoretic mobility shift assay (EMSA) was performed with rCosR to determine if CosR directly binds to the promoter regions of *sodB*, *dps*, *luxS* and *ahpC*. The results showed that rCosR bound to the promoter regions, and binding of rCosR to the DNA resulted in concentration-dependent mobility shift (Figure 5B). Unlabeled target DNA fragments effectively competed with the labeled DNA probes of *sodB*, *dps*, *luxS* and *ahpC*, but the internal coding regions did not (Figure 5B), indicating specific binding of CosR to the target promoter regions. These results demonstrate that CosR is a pleiotropic regulator, particularly modulating the expression of oxidative stress genes in *C. jejuni*.

**Table 1:** Identification and expression change of protein spots in 2DGE at different CosR levels.

Spotno.^a^	Geneno.	Description of proposed function	Protein	Fold change^b^		*p*I	M.W(kDa)
				CosR knockdown	CosR overexpression		
**Metabolism and biosynthesis**							
1	*cj0287c*	transcription elongation factor	GreA	D:1.9	U:3.0	4.9	18.0
2	*cj0640c*	aspartyl-tRNA synthetase	AspS	D:2.1	U:2.5	5.8	66.7
3	*cj0918c*	ribose-phosphate pyrophosphokinase	PrsA	D:2.4	U:2.4	7.7	33.8
4	*cj0932c*	phosphoenolpyruvate carboxykinase	PckA	D:2.1	U:3.0	5.8	59.5
5	*cj0473*	transcription antitermination protein	NusG	U:1.5	D:2.6	6.2	20.2
6	*cj0543*	prolyl-tRNA synthetase	ProS	U:2.1	D:2.2	5.9	65.1
7	*cj0994c*	ornithine carbamoyltransferase	ArgF	U:1.4	D:2.2	6.2	35.1
**Copper tolerance**							
8	cj1516	probable periplasmic oxidoreductase	CeuO	U:1.2	D:2.0	8.6	59.1
**Stress response**							
9	*cj0334*	alkyl hydroperoxide reductase	AhpC	D:2.0/1.7	U:2.9/2.9	5.7	22.0
10	*cj0012c*	rubrerythrin, non-haem iron protein	Rrc	U:1.4/1.7	D:1.7/1.5	5.5	25.0
11	*cj1534c*	putative bacterioferritin	Dps	U:2.8/2.0	D:3.1/2.9	5.7	17.2
12	*cj0169*	superoxide dismutase	SodB	U:2.0	D:2.6	5.8	25.0
**Heat shock**							
13	*cj1221*	chaperonin GroEL	GroEL	D:2.2	U:3.1	4.9	58.0
14	*cj0518*	heat shock protein 90	HtpG	U:1.4	D:2.5	5.0	69.7
15	*cj1260c*	chaperone DnaJ	DnaJ	U:1.6	D:2.8	6.1	42.0
**Signal transduction**							
16	*cj1110c*	probable MCP-type signal transduction protein	Cj1110c	U:1.7	D:2.1	6.0	48.5
17	*cj1189c*	bipartite energy taxis response protein	CetB	U:1.7/1.7	D:2.6/2.4	8.0	19.3
**Regulation**							
18	*cj0355c*	two-component regulator	CosR	D:3.0/3.3/3.1	U:2.7/2.7/3.5	5.3	25.5
19	*cj0440c*	putative transcriptional regulator	Cj0440c	U:1.7	D:2.0	5.6	26.1
20	*cj1198*	S-ribosylhomocysteinase	LuxS	U:1.7	D:2.2	6.5	18.2
21	*cj1227c*	putative two-component regulator	CprR	U:1.5	D:3.2	5.6	25.5
**Electron transport**							
22	*cj0074c*	iron-sulfur cluster binding protein	Cj0074c	D:1.9	U:2.2	6.5	54.9
23	*cj0537*	2-oxoglutarate-acceptor oxidoreductase subunit OorB	OorB	D:3.5	U:3.4	7.8	31.2
**Unknown**							
24	*cj0170*	hypothetical protein	Cj0170	D:1.5/2.2	U:3.4/3.2	7.6	28.7
25	*cj0898*	putative histidine triad (HIT) family protein	Cj0898	D:2.1	U:2.6	6.2	14.0
26	*cj1659*	periplasmic protein p19	p19	D:1.9	U:2.9	5.2	19.6
27	*cj0771c*	putative NLPA family lipoprotein	Cj0771c	U:1.7	D:2.6	8.7	28.9
28	*cj0772c*	putative NLPA family lipoprotein	Cj0772c	U:1.2	D:2.8	7.8	28.6
29	*cj1419c*	putative methyltransferase	Cj1419c	U:1.6	D:2.5	6.0	29.8
30	*cj1420c*	putative methyltransferase	Cj1420c	U:2.2	D:1.6	6.3	29.8
31	*cj1426c*	putative methyltransferase family protein	Cj1426c	U:2.3	D:1.8	6.0	33.6
32	*cj1548c*	putative NADP-dependent alcohol dehydrogenase	Cj1548c	U:1.5	D:2.4	6.7	39.5
33	*cj1626c*	putative periplasmic protein	Cj1626c	U:1.7	D:2.8	5.5	15.4

a The spot number corresponds to that indicated in Figure 4. Fold changes of multiple protein spots are separated by a slash in the same order from left to right as Figure 4.

b ‘D’ and ‘U’ mean ‘downregulation’ and ‘upregulation’, respectively.

### Determination of CosR binding sequence

**Figure 6:**
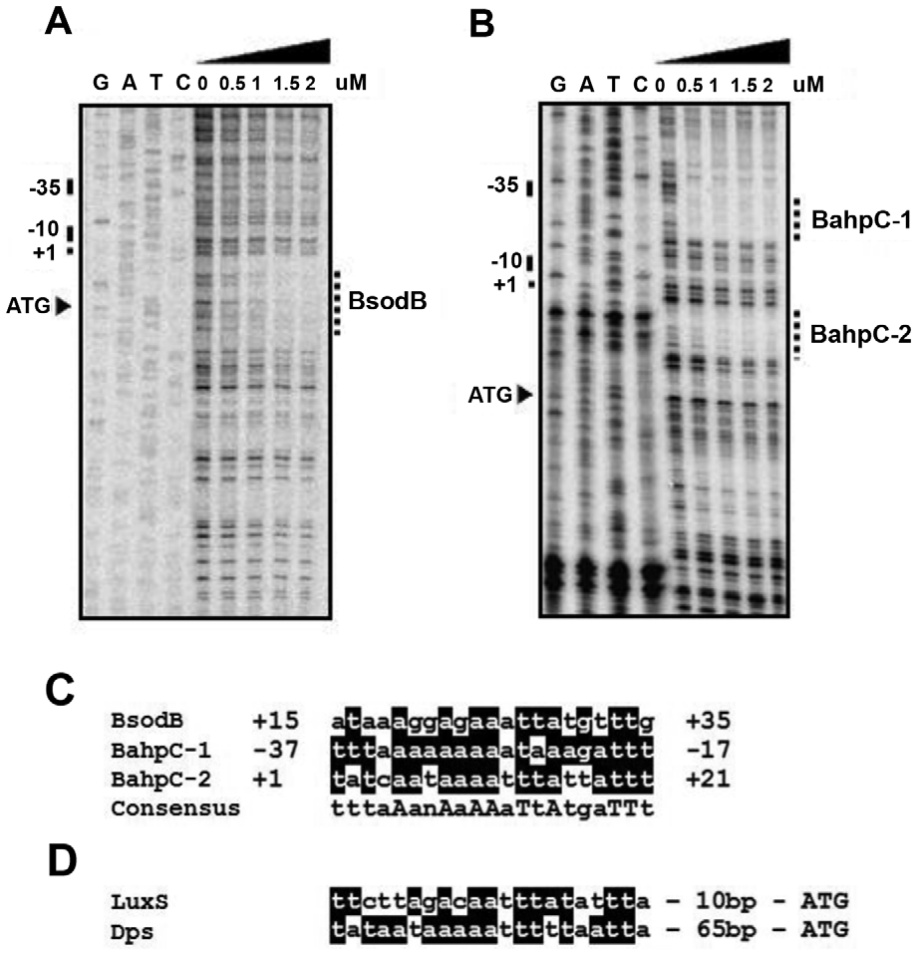
DNase I footprinting of the *sodB* and *ahpC* promoter regions. The CosR binding sites in the promoter regions of *sodB* (A) and *ahpC* (B) were determined by DNase I footprinting assays. Based on previous studies [6], [9], the start codon (ATG), transcriptional start site (+1), and −10 and −35 regions of *sodB* and *ahpC* are indicated on the left. The CosR binding sites are indicated with dotted lines and labeled “BsodB” for the binding site in the *sodB* promoter and “BahpC-1 and BahpC-2” for the two binding sites in the *ahpC* promoter. (C) Alignment of CosR binding sequences for *sodB* and *ahpC*. Nucleotide sequences of the CosR binding sites determined from panel (A) and (B) were compared. (D) Prediction of putative CosR binding sites in the regulatory region of *dps* and *luxS* genes based on homology to the CosR-binding sequence described in panel (C). Highly conserved nucleotides are shaded on black background, and identical nucleotides are indicated in capital letters.

Among the oxidative stress proteins identified by 2DGE, CosR negatively and positively regulated SodB and AhpC, respectively (Figure 5A), and bound to the promoter region of the encoding genes (Figure 5B). DNase I footprinting assays were performed to determine the CosR binding sites in the promoter region of *sodB* and *ahpC* genes. DNase I footprinting results were interpreted based on the previous studies of the *sodB* and *ahpC* promoter sequences in *C. jejuni* [6], [9]. The results showed a region immediately downstream of the −10 region of the *sodB* promoter, which spans the leader sequence and the start codon of *sodB*, was protected from DNase I treatment (Figure 6A). Two CosR binding sites were found in the *ahpC* promoter, including the −37 to −17 region overlapping the −35 region of the *ahpC* promoter and the +1 to +21 region that is between the −10 region and the *ahpC* start codon (Figure 6B). The nucleotide sequences of the three CosR binding sites obtained from the DNase I footprinting assays were aligned to predict the consensus CosR binding sequence (Figure 6C). The alignment revealed a 21-bp CosR-binding sequence (tttaAanAaAAaTtAtgaTTt, ‘n’ represents any nucleotide, lowercase letters indicate less conserved residues, and capital letters are highly conserved residues; Figure 6C), suggesting that CosR binds to a specific target site in the CosR-regulated genes. Putative CosR binding sites were also identified in the regulatory region of *dps* and *luxS* genes based on homology to the CosR-binding sequence (Figure 6D).

### Regulation of SodB by CosR

**Figure 7:**
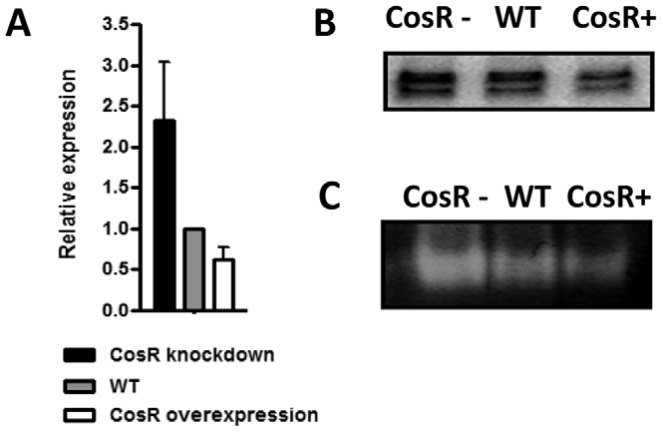
Negative regulation of *sodB* by CosR. (A) qRT-PCR analysis of *sodB* transcription. Results are expressed as the mean and standard deviation from three independent experiments. (B) *sodB* transcription determined by primer extension assays at different CosR levels. The doublet bands show two *sodB* transcriptional start sites in *C. jejuni*, and the top band is the major. “CosR−” and “CosR+” indicate CosR knockdown and CosR overexpression, respectively. (C) Enzymatic activity of SodB at different CosR levels. In these experiments, CosR knockdown was achieved with 1.5 µM CosR-PNA. These results show the representative of three independent experiments.

Although SodB is a key enzyme contributing to the detoxification of O_2_^−^, its regulation has not been well understood in *C. jejuni*. Since the results of 2DGE, EMSA and DNase I footprinting assays indicated that CosR may regulate *sodB* transcription (Figures 4, 5, and 6A), qRT-PCR and primer extension assays were performed to further confirm the regulation of *sodB* transcription by CosR. In both assays, antisense inhibition of CosR upregulated *sodB* transcription, whereas CosR overexpression reduced the level of *sodB* transcription compared to the wild type (Figures 7A and 7B). To examine if the changes in SodB expression would affect its enzyme activity, the superoxide dismutase colorimetric assay was performed. Consistently, the superoxide dismutase activity was enhanced by CosR knockdown and attenuated by CosR overexpression (Figure 7C). These results demonstrated that CosR regulates *sodB* transcription and affects the SodB enzyme activity.

### Effect of CosR on oxidative stress resistance

**Figure 8:**
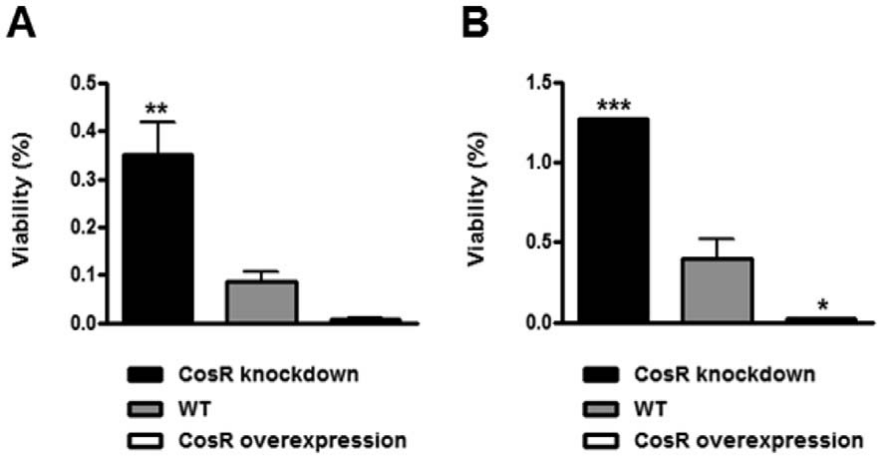
Effect of CosR on oxidative stress resistance. Changes in the viability of *C. jejuni* by paraquat (A) and hydrogen peroxide (B); 1 mM each oxidant for 1 hr exposure. Viability is indicated by percent change in viable count before and after each oxidant treatment. CosR knockdown was achieved with 1.5 µM CosR-PNA. Results show the average and standard deviation of triplicate samples from a single experiment. The experiment was repeated three times with similar results. *: *P*<0.05; **: *P*<0.01; ***: *P*<0.001. The *P* values are the results of one-way ANOVA analysis of variance with Dunnett's post-tests at a 95% confidence interval using Prism software (version 5.01; GraphPad Software Inc., USA).

Because CosR regulates several important oxidative stress genes in *C. jejuni*, susceptibility to oxidative stress was measured at different CosR levels. Viable counts were determined before and after 1 hr exposure to oxidant. The *C. jejuni* strain overexpressing CosR was more susceptible to paraquat and H_2_O_2_ compared to the wild type, whereas CosR knockdown by antisense rendered *C. jejuni* more resistant to oxidative stress (Figure 8). These results showed that CosR significantly contributes to the oxidative stress resistance of *C. jejuni*.

## Discussion

Given the limited presence of only three sigma factors in *C. jejuni*, the 12 two-component response regulators are thought to be important for the gene regulation of this bacterium [13]. Indeed, several two-component response regulators in *C. jejuni* have been reported to significantly contribute to the virulence features of this bacterial pathogen, such as bacterial motility, colonization and bile resistance [14], [16], [17], [34]. Among the 12 response regulators, the response regulator CosR is known to be essential for the viability of *C. jejuni* [17], [20], suggesting that CosR may regulate critical cellular processes in *C. jejuni*. In this study, the results of 2DGE analysis suggested that CosR is a pleiotropic regulator involved in various cellular processes, such as macromolecule biosynthesis, metabolism, gene regulation and oxidative stress response (Figure 4 and Table 1). Interestingly, CosR regulated the expression of several important oxidative stress proteins in *C. jejuni*, including Dps, LuxS, Rrc, AhpC and SodB (Figures 4, 5A, and Table 1). Dps is an iron-binding protein contributing to oxidative stress defense by sequestering free intracellular iron available for the Fenton reaction, and its mutation makes *C. jejuni* more susceptible to H_2_O_2_ than the wild type [8]. LuxS is S-ribosylhomocysteinase and its mutation downregulates *ahpC* and *tpx* in the presence of H_2_O_2_ by an unknown mechanism, rendering *C. jejuni* susceptible to H_2_O_2_ and cumene hydroperoxide [35]. Rrc is a non-heme iron protein that is extremely sensitive to reactive oxygen species [36]. AhpC confers resistance to cumene hydroperoxide [6], [10]. Notably, CosR regulated SodB, a key enzyme of oxidative stress resistance in *C. jejuni*. Unlike other bacteria that often possess multiple forms of superoxide dismutase (SOD), such as Mn-cofactored SOD (SodA), Fe-cofactored SOD (SodB), and Cu and Zn-cofactored SOD (SodC) in *E. coli* [5], *C. jejuni* harbors only SodB [9]. As the sole SOD of *C. jejuni*, SodB plays an important role in *C. jejuni*'s resistance to oxidative stress and survival in various conditions such as foods, chicken guts and epithelial cells [9], [10]. In this study, we demonstrated that CosR negatively regulates *sodB* expression. CosR bound to a region downstream of the −10 element of the *sodB* promoter (Figure 6A). In many other bacteria, Fur (ferric uptake repressor) is directly or indirectly involved in SOD regulation. For example, Fur downregulates *sodA*, but upregulates *sodB* through suppression of the small regulatory RNA RyhB that inhibits *sodB* translation in *E. coli* [37]–[39]. Even in the closely related bacterium *H. pylori*, Fur regulates *sodB* expression by directly binding to its promoter [40]. However, a *fur* mutation in *C. jejuni* does not affect SodB activity [41], suggesting *C. jejuni* may possess unique mechanisms for *sodB* regulation. To the best of our knowledge, CosR is the first regulator shown to control *sodB* expression in *C. jejuni*.

Among the 32 CosR-regulated proteins identified in this study, 12 proteins were positively regulated by CosR (Figure 4 and Table 1). To address if CosR binds to the positively regulated genes, we performed EMSA and DNase I footprinting assays targeting *ahpC*, which is the sole oxidative stress gene positively regulated by CosR in this study. EMSA demonstrated that CosR bound to the *ahpC* promoter (Figure 5B) and DNase I footprinting assays identified two CosR binding sites in the *ahpC* promoter region (Figure 6B). Whereas *ahpC* is often regulated by the peroxide-sensing activator OxyR in many bacteria [5], [42], *C. jejuni* lacks OxyR; instead, PerR (peroxide regulon repressor) negatively regulates *ahpC* expression in *C. jejuni* [41]. Due to the iron-dependence of PerR, *ahpC* expression is suppressed in iron-rich conditions [6], [41]; however, overall mechanisms for *ahpC* regulation remain unknown in *C. jejuni*. It would be interesting to carry out future studies to examine how PerR and CosR may coordinately regulate *ahpC* expression in *C. jejuni*.

In this work, we demonstrated that CosR binds to the promoter regions of *sodB* and *ahpC* and regulates their expressions differentially. At this point, the molecular mechanism of differential regulation by CosR remains unexplained; however, the regulation mechanisms of OmpR reported in other bacteria may provide valuable insight into this OmpR-type response regulator in *C. jejuni*. In *E. coli* and *Salmonella*, the function of OmpR is influenced by phosphorylation, since phosphorylation significantly affects OmpR binding to DNA and its interaction with OmpR and RNA polymerase [43]–[46]. As a global regulator in *E. coli*, OmpR is reported to modulate expression of 125 genes positively and negatively [47]. In association with the sensor kinase EnvZ, particularly, OmpR regulation of major porin genes *ompC* and *ompF* may be the most well-defined regulatory role played by this response regulator. EnvZ has both kinase and phosphatase activities, phosphorylating OmpR at high medium osmolarity and dephosphorylating the phosphorylated form (OmpR-P) at low osmolarity. Four (F1, F2, F3, and F4) and three (C1, C2, and C3) OmpR binding sites are present in the promoters of *ompF* and *ompC*, respectively. At high osmolarity, OmpR-P binds to the upstream site F4 and interacts with OmpR-P in F1, F2 or F3, leading to formation of a loop by OmpR-OmpR interactions and thereby repressing *ompF*, whereas binding of OmpR-P to C2 and C3 at high osmolarity promotes interactions with RNA polymerase and activates *ompC* [48]. As shown in the porin gene regulation in *E. coli*, OmpR negatively and positively regulates porin genes depending on osmolarity and phosphorylation. In *Salmonella*, the mechanism of OmpR regulation has been relatively less extensively investigated compared to *E. coli*. OmpR activates SsrAB, a two-component regulatory system regulating the expression of the type III secretion system of *Salmonella* pathogenicity island 2 (SPI2) by binding to the promoter regions of *ssrAB* [49], [50]. While the unphosphorylated OmpR binds to only a single spot in the *ssrA* promoter, OmpR-P binds to multiple sites in the *ssrA* and *ssrB* promoters [49]–[51]. As reflected in OmpR regulation of *ssrAB* and porin genes, the regulation mechanism of OmpR cannot be explained without considering phosphorylation. If phosphorylation influences the binding affinity of CosR to the target promoters as OmpR, the phosphorylated form of CosR may bind to more spots in the *sodB* and *ahpC* promoters than identified in this work. Similar to OmpR, presumably, CosR phosphorylation would affect interactions with RNA polymerase and/or the CosR protein in the vicinity, either activating or repressing gene expression. This model may explain differential regulation of *sodB* and *ahpC* by CosR; however, all these possibilities can be proved after investigating the following research questions, such as if CosR is phosphorylated, whether CosR is an orphan response regulator or CosR has its cognate sensor kinase, how phosphorylation affects CosR binding to target promoters, and if CosR interacts with RNA polymerase and the CosR protein. These series of studies still await future investigations.

A previous proteomic study reported that the level of CosR was reduced by paraquat (O_2_^−^) [20]. Consistently, western blot analysis in this study confirmed that the CosR level was significantly decreased by exposure to paraquat (Figure 2A). However, H_2_O_2_ did not change the CosR level (Figure 2B), suggesting CosR may respond selectively to O_2_^−^ stress. CosR repression by antisense significantly affected the viability of *C. jejuni* (Figure 3B); however, the level of CosR was not reduced by exposure to H_2_O_2_ despite substantial viability changes (Figure 2B). These results suggest that killing of *C. jejuni* under oxidative stress is not necessarily mediated by CosR depending on the type of oxidative stress, though CosR is essential to the viability of *C. jejuni*. The CosR reduction in response to O_2_^−^ stress would derepress the oxidative stress genes that are negatively regulated by CosR, such as *sodB* and *dps*. Consequently, *sodB* derepression may confer resistance to O_2_^−^ stress because SodB converts O_2_^−^ to H_2_O_2_. As the sole superoxide dismutase present in *C. jejuni*, SodB is the only enzyme able to detoxify O_2_^−^ in *C. jejuni*. In addition, the derepression of Dps by CosR reduction may enhance the sequestration of free iron and reduce the production of hydroxyl radicals generated from O_2_^−^ and H_2_O_2_ by the Fenton and Haber-Weiss reactions. In fact, there is no enzyme capable of detoxifying hydroxyl radicals, though hydroxyl radicals can damage most biomolecules [5]. In contrast to SodB and Dps, the AhpC level will be decreased by CosR reduction, because AhpC is positively regulated by CosR. The opposite regulatory patterns of SodB and AhpC, observed in this study, have often been reported in *C. jejuni*. Recently, Svensson *et al.* demonstrated that a mutation of the *cprS* sensor kinase upregulated AhpC but downregulated SodB [18]. According to a proteomic analysis performed by Holmes *et al*. [52], AhpC was significantly upregulated in iron-limited conditions, whereas SodB was downregulated. The differential regulation of SodB and AhpC would achieve efficient defense against various stresses of different reactive oxygen species, though its molecular mechanism has not been understood. As key enzymes involved in oxidative stress resistance, AhpC and SodB act on different substrates. While SodB consumes O_2_^−^, AhpC detoxifies organic hydroperoxides and can scavenge only low physiological concentrations of H_2_O_2_ [53]; thus, AhpC provides resistance to organic hydroperoxides but not to H_2_O_2_ in sensitivity tests in *E. coli* [53], [54]. Similarly, *ahpC* mutations change the susceptibility of *C. jejuni* to neither menadione (a O_2_^−^ generator) nor H_2_O_2_ [6], [10]. The level of CosR protein was decreased by exposure to O_2_^−^ (Figure 2A), and subsequently the CosR reduction would derepress the expression of SodB and Dps, which are important oxidative stress defense proteins to overcome the imposed O_2_^−^ stress, but may reduce the expression of AhpC that cannot contribute to the detoxification of O_2_^−^.

The overall role of CosR in oxidative stress resistance was evaluated by performing sensitivity assays at different CosR levels. Results of these sensitivity assays demonstrated that the CosR knockdown by antisense increased resistance to both O_2_^−^ and H_2_O_2_, whereas CosR overexpression decreased the resistance (Figure 8). The effect of CosR on the oxidative stress resistance of *C. jejuni* would result from cumulative changes in multiple oxidative stress resistance factors regulated by CosR; CosR knockdown upregulates the expression of a few oxidative stress resistance proteins. Additionally, SodB is known to confer resistance to both peroxide (e.g. H_2_O_2_ and cumene hydroperoxide) and O_2_^−^ (e.g. manadione) in *C. jejuni* [9], [10], whereas AhpC and KatA are involved in defense only against cumene hydroperoxide and H_2_O_2_, respectively [10]. Therefore, the upregulation of SodB by CosR knockdown may contribute to the resistance to both paraquat and H_2_O_2_ (Figure 8). Iron participates in the Fenton reaction and plays an important role in generating reactive oxygen species; thus, the iron uptake regulator often regulates oxidative stress defense genes, and oxidative stress regulators also control the expression of genes involved in iron homeostasis [10], [52]. To investigate if CosR may affect iron levels in *C. jejuni*, we determined intracellular iron levels using Ferene-S, a chromogenic iron chelator [55], [56]. The results showed that both CosR knockdown and overexpression did not change intracellular iron levels (data not shown); however, this does not mean that CosR is functionally independent of other iron regulators. Since the Fur regulon proteins p19 and AhpC were downregulated in the CosR knockdown condition (Table 1), CosR likely interacts with Fur directly or indirectly. This possibility needs to be examined in future research.

CosR homologs are found primarily in the ε-proteobacteria, such as *Helicobacter* and *Wolinella* (Figure 1A). CosR exhibits high (60%) amino acid sequence similarity to *H. pylori* HP1043 and other homologs (Figure 1A). Interestingly, HP1043 is also one of two essential two-component response regulators in the closely related bacterium *H. pylori* [57], [58]. CosR expressed under the control of the HP1043 promoter can substitute for HP1043 in *H. pylori* [59], suggesting functional similarity between these two proteins. Compared to other CosR homologs, however, HP1043 has unique differences particularly in its N-terminus, including the substitution for and deletion of highly conserved aspartate residues, which are potential sites of phosphorylation (Figure 1A). The alteration of these residues suggests that phosphorylation of HP1043 may differ from that of other CosR homologs. In fact, HP1043 is not phosphorylated by the small molecule phosphodonor acetyl phosphate *in vitro* and is known to have a phosphorylation-independent action [23], [60]. Although it has not been understood if CosR is phosphorylated, the conformational heterogeneity of CosR observed in the 2DGE analysis suggests that CosR may be phosphorylated within *C. jejuni* (Figures 5A). Since no potential histidine kinase was identified near the *cosR* gene, it remains unknown whether CosR would be phosphorylated by a yet-unidentified histidine kinase or by low molecular weight phosphodonors such as acetyl phosphate in *C. jejuni*. Acetyl phosphate is the intermediate produced in the enzymatic conversion between acetyl coenzyme A and acetate, and can autophosphorylate a number of two-component response regulators [61]. According to the genome sequence, *C. jejuni* possesses *pta* (Cj0688) and *ackA* (Cj0689), encoding phosphotransacetylase (Pta) and acetate kinase (AckA), respectively [13], which are responsible for the enzymatic reactions producing acetyl phosphate. Details of CosR phosphorylation will be investigated in future studies.

To characterize essential genes, conditional mutants have been constructed in *E. coli*, *Salmonella*, and *Helicobacter* [62]–[64]; however, construction of conditional mutants is not technically feasible in *C. jejuni*, because tools for genetic manipulation are less well developed in this fastidious bacterium. Thus, as a novel approach to overcome this technical limitation in investigating an essential gene, antisense-mediated gene silencing was used in this study to knockdown the expression of CosR. Antisense technology is a useful genetic tool in eukaryotic systems, but is seldom used in prokaryotic studies [26], [27]. Jeon and Zhang recently reported that antisense successfully controlled the expression of *C. jejuni* CmeA, a periplasmic component of the CmeABC efflux pump [30]. In the present study, 8-hr PNA treatment suppressed CosR expression (Figure 3A), but treatment longer than 12 hrs reduced the effect on CosR knockdown, as assessed by western blotting (data not shown). Considering the high stability of PNA, these findings may be due to significant increase in bacterial population, which may reduce the ratio of PNA to bacteria. For that reason, application of antisense technology in prokaryotes should consider rapid bacterial growth compared to relatively slowly growing eukaryotic cells. High CosR-PNA concentrations significantly reduced the viability of *C. jejuni*, confirming the essentiality of CosR for bacterial survival (Figure 3B). As expected, CosR is involved in the regulation of critical metabolic pathways in *C. jejuni*. The results of 2DGE demonstrated that PckA, an essential enzyme of central carbon metabolism, was positively regulated by CosR (Figure 4 and Table 1). *C. jejuni* lacks 6-phosphofructokinase and is therefore unable to utilize exogenous glucose to produce pyruvate [13]. Instead, pyruvate is supplied by other metabolites that are primarily derived from amino acids. PckA converts oxaloacetate to phosphoenolpyruvate, which is then converted to pyruvate by pyruvate kinase [32]. Because *C. jejuni* does not have phosphoenolpyruvate synthase, PckA is the only way to produce phosphoenolpyruvate, and is thus essential for *C. jejuni* growth [32]. Although it has not been understood whether CosR regulates PckA directly or not, this regulation may partly explain why CosR is essential for *C. jejuni* viability. CosR also regulated CprR, another essential response regulator in *C. jejuni*. While the role of CprR has not been elucidated due to the inability to construct its mutant, its cognate sensor kinase CprS was shown to be involved in biofilm formation, oxidative stress resistance, and chicken colonization [18]. Interestingly, a CprS mutation decreased CosR expression, suggesting a functional relationship between these two essential response regulators; however, detailed mechanisms for sustaining the viability of *C. jejuni* by CosR still remain largely unknown.

In the present study, our findings provided new insights into the regulatory mechanisms for oxidative stress resistance in *C. jejuni*, an important human pathogen, clearly demonstrating that CosR regulates the expression of several key oxidative stress genes, particularly *sodB*, which is the sole superoxide dismutase gene in *C. jejuni*. In addition, we showed that antisense-mediated gene silencing is a useful approach to investigate essential genes in bacterial species in which genetic tools have not been well developed. To our knowledge, this is the first proteomic study to characterize an essential regulator in bacteria using antisense. The same technical approach will be useful to the investigation of essential genes in *C. jejuni* and other bacteria as well.

## Materials and Methods

### Bacterial strains and culture conditions

*C. jejuni* NCTC 11168, the first genome-sequenced *Campylobacter* strain, was used for this study [13]. *C. jejuni* was grown at 42°C on Mueller-Hinton (MH) media (Difco) under microaerobic conditions generated by Anoxomat™ (Mart Microbiology B.V, Lichtenvoorde, Netherlands). Culture media were occasionally supplemented with kanamycin (50 µg ml^−1^) or chloramphenicol (10 µg ml^−1^) where required. Broth cultures were microaerobically grown with shaking at 180 rpm after adjusting the absorbance at 600 nm to approximately 0.07.

### Preparation and treatment of CosR-PNA

The PNAs used in this study were conjugated with the oligopeptide (KFFKFFKFFK) to improve the permeability of PNA into bacterial cells [29], and were commercially synthesized by Panagene (Daejeon, Korea). Since the start codon and Shine-Dalgarno motif are known to be the primary target for antisense inhibition [65], a 16-mer PNA (CATTTGTTCTATCCTT) was designed to reverse-complementarily bind to the leader sequence spanning the ribosomal binding site and the start codon of *cosR* based on the genome sequence of *C. jejuni* NCTC 11168 [13]. Another PNA oligomer (Cont-PNA; TATCTCTCTCTGTATT), which possesses the same number of bases as CosR-PNA but does not exhibit significant homology to *cosR* and other *C. jejuni* genes, was used as a control. *C. jejuni* was treated with PNA as described previously by Jeon and Zhang [30]. Briefly, an overnight culture of *C. jejuni* on MH agar plates was collected and resuspended in MH broth to an optical density of approximately 0.07 at 600 nm (approximately 1×10^7^ CFU ml^−1^). PNAs were added to broth cultures and *C. jejuni* was grown for 8 hrs under the culture conditions described above.

### Construction of a CosR-overexpressing strain

A *C. jejuni* strain that overexpresses *cosR* was constructed by chromosomal integration of an extra copy of *cosR* using the previously-reported methodology of chromosomal integration of a gene into a non-coding spacer region of rRNA gene clusters [66]. Briefly, pFMB is a pUC19 derivative carrying an rRNA gene cluster and a kanamycin resistance cassette, and was constructed according to the method described by Karlyshev and Wren [66]. A DNA fragment containing *cosR* and its upstream flanking region was amplified with CosRF_Xba and CosRR_Xba primers (Table 2). The PCR product was digested with XbaI and cloned into pFMB. The constructed plasmid was delivered to the bacterial cell by electroporation, and quantitative real time polymerase chain reaction (qRT-PCR) and western blotting was performed to confirm the increased expression of CosR.

**Table 2:** Primers used in this study.

Primer	DNA sequence from 5′ to 3′
CosRF_Xba	CCCTTGAAGAGTCTAGAGACTTTGTAAGCTT
CosRR_Xba	CAAGCATCTAGACATACGCAGTCTTTTGTAA
CosRHis(NdeI)-F	GGATAGAACATATGAGAATTTTAGTTAT
CosRHis(BamHI)-R	GCACCGAAGGATCCAAATTGTTAAGA
SodB_F_PriEx	GCTTGGAGTATTAGCTGCAAAAACATTA
SodB_R_PriEx	CTTACA AGATCTTTACCTGCAAATTCAGTAT
SodB_F	GCGAAGGATCCTAGTAATGCTGAGATTAGTA
SodB_R	CAGCACTCTAGAAATCACCAAAAGCATTGGTAT
Dps_F	ACCTGGTGTGGAAGTTGGAAAGTTGATC
Dps_R	ATCAAAAAGTTCTGCCATTTCTTCATAAGC
LuxS_F	AAAGCTCCTGGTAAGGCCAAACAAGTG
LuxS_R	ACGCAAATCAAATACGCTAATATCATCACCC
AhpC_F	ATCATCAACGATAGCATTCACTGGACAC
AhpC_R	AATACTACCGCTCCTTTTGGACCTATG
SodB_IC_F	TGCTTTTGGTGATTTTTTGAGTGCTGAAAC
SodB_IC_R	ATAAAAGCTAACTGATCCCATGCCTTC
Dps_IC_F	TTATGAAGAAATGGCAGAACTTTTTGATAG
Dps_IC_R	ACCTTGTAAAGTAGCGCCTATCATCC
Luxs_IC_F	TAAAGACATTATGAGCGAAAAAGGTACTC
Luxs_IC_R	TTTTTGGGCAATTTGTTTGGCTTCATCTAAAG
Ahpc_IC_F	ACTCCAGTAAATCAAGGTGGTATTGGTC
Ahpc_IC_R	TTTTTGCCAAGATATTCAGCCACGCC
AhpC_FP_F	ACTAGCACAAGCTGGTTGATCATTATG
AhpC_ES_R	AATACTACCGCTCCTTTTGGACCTATG
Cjr01-F	TGCTAGAAGTGGATTAGTGG
Cjr01-R	GTATTAGCAGTCGTTTCCAA
sodB-RT-F	AGCTATCATCATGGAAAACA
sodB-RT-R	GATCTTTACCTGCAAATTCA

Underlines indicate enzyme recognition sites.

### Purification of rCosR

rCosR was produced in *E. coli* BL21 (DE3) with the pET15b vector (Novagen). The *cosR* gene was PCR-amplified using primers CosRHis(NdeI)-F and CosRHis(BamHI)-R (Table 2). After digestion with NdeI and BamHI, the PCR product was cloned into pET15b that had been digested with the same enzymes. *E. coli* BL21 (DE3) carrying plasmid pET15b::*cosR* was grown to an optical density of approximately one at 600 nm. After induction with 0.5 mM IPTG for 3 hrs, rCosR was purified under the native conditions using Ni^2+^ affinity chromatography. The purified rCosR was used to generate polyclonal antibodies against rCosR in rabbits commercially by AbFrontier (Seoul, Korea).

### SDS-PAGE and western blotting

After growing for 8 hrs in antisense experiments, bacterial cells were disrupted by sonication and insoluble cell debris was removed by centrifugation at 10,000× *g* for 10 min. Bacterial samples under oxidative stress were prepared by following the same culture conditions used in the oxidative sensitivity test (5 hr culture and 1 hr exposure to oxidative stress agents). After adjusting the sample amount by the Bradford assay (BioRad), the equal amount of protein was boiled for 3 min in the loading buffer (0.05 M Tris–HCl pH 8, 1.6% SDS, 25% glycerol, 5% 2-mercaptoethanol, 0.003% bromophenol blue). After electrophoresis in a 12% polyacrylamide gel in Tris-Glycine buffer, gels were either stained with Coomassie Blue or blotted to PVDF membrane (Immobilon-P, Millipore). The blotted membrane was incubated in blocking solution (Tris-buffered saline with 5% skim milk and 0.05% Tween 20) for 1 hr and was probed with the primary antibody (rabbit anti-rCosR, 1∶200 dilution) for 1 hr. After three washings, the membrane was incubated with the secondary antibody (goat anti-rabbit IgG-HRP, 1∶5,000, Santa Cruz Biotech.). After washing, the signals were developed with WEST-ZOL plus western blot detection system (Intron Biotechnology, Korea).

### Proteomic analysis by 2DGE

A 2DGE analysis was performed according to a method reported previously [67]. *C. jejuni* cells were grown in MH broth, which was occasionally supplemented with 1.5 µM CosR-PNA for CosR knockdown, to the mid-exponential phase for approximately 8 hrs with shaking and harvested by centrifugation at 3,000× g for 15 min. The pellet was resuspended and incubated in sample preparation solution (7 M urea, 2 M thiourea, 4% CHAPS, 0.5% IPG buffer, and 40 mM DTT) for 5 min. Following centrifugation at 32,000× g for 20 min at 4°C to remove insoluble proteins and large macromolecular complexes, the supernatant was precipitated with 3× volume of 100% acetone for 2 hrs at −20°C. After additional centrifugation, the protein pellet was resuspended in rehydration buffer (7 M urea, 2 M thiourea, 2% CHAPS, 0.5% IPG buffer, 0.02% bromophenol blue). Protein concentration was determined by the Bradford assay (BioRad). For the first-dimension separation, 200 µg of protein from each sample was separated by isoelectric focusing using 13 cm immobilized p*I* gradient strips ranging from 3 to 10 (GE Healthcare). For the second dimensional separation, proteins were separated according to their molecular weight by SDS-PAGE using 12% acrylamide gels. Gels were visualized by Colloidal Coomassie Blue staining (Invitrogen). The protein expression profiles were analyzed from the multiple gel images with Progenesis SameSpots (Nonlinear Dynamics). Though the 2-fold cut-off is often used in 2DGE analysis, 1.5-fold cut-off was adopted in this study, since the protein expression changes of this work resulted from knockdown, not from complete gene inactivation by gene knockout. A 1.5-fold change was set as a threshold during image analysis to determine proteins of different expression levels. Protein spots showing different expression levels were excised from stained gels and followed by in-gel digestion according to Mann's method [68]. Gel slices were washed twice with water and acetone-water (1∶1) solution. To shrink gel pieces, acetonitrile was added and washed with 0.1 M NH_4_HCO_3_. After removal of all liquid by a Speedvac concentrator, gel pieces were reduced and alkylated with 10 mM DTT and 55 mM iodoacetamide, respectively. The gel pieces were dried again and the protein was digested by trypsin at 37°C overnight in digestion buffer (50 mM NH_4_HCO_3_, 5 mM NaCl, 12.5 mg ml^−1^ trypsin). The volume of the supernatant containing the generated peptides was reduced by the Speedvac concentrator. Peptide mass fingerprinting was done with a Voyager-DETM STR Biospectrometry Workstation (PerSeptive Biosystems, Framingham). Proteins were identified by searching for peptide mass fingerprints with the programs MS-FIT (UCSF Mass Spectrometry Facility at http://prospector.ucsf.edu/prospector/mshome.htm) and MASCOT (Matrix Science, London, UK at http://www.matrixscience.com) supplemented with an option for *Campylobacter* in the NCBI database.

### qRT-PCR

Total RNA was isolated using the RNeasy Mini Kit (Qiagen) from *C. jejuni* cultures incubated as mentioned above in the proteomics analysis. After DNase treatment of the total RNA solution using the Turbo DNA-free™ kit (Ambion), cDNA was synthesized using the Omniscript RT kit (Qiagen) and random hexamers (Invitrogen). Quantification of cDNA was carried out using IQ SYBR Green PCR Supermix (Bio-Rad), and real-time amplification of PCR product was analyzed using the iCycler™ Optical Module (Bio-Rad). The amplification program consisted of one cycle at 95°C for 5 min, followed by 40 cycles at 95°C for 30 sec, 50°C for 30 sec, and 72°C for 30 sec. mRNA levels of *sodB* were normalized to the expression level of *Cjr01* encoding 16s rRNA. The relative amount of cDNA was calculated according to the 2^−ΔΔC^_T_ method [69]. The sequences of the *sodB* (sodB-RT-F and sodB-RT-F) and *Cjr01* (Cjr01-F and Cjr01-R) primers are presented in Table 2.

### Primer extension assay

Primer extension assays were performed as described previously [70]. Briefly, *C. jejuni* was grown in MH broth under the same conditions described in the proteomics analysis. The total RNA was purified with Trizol reagent (Invitrogen) according to the manufacturer's instructions. Purified RNA was resuspended in sterile distilled RNase-free water and the RNA concentration was determined by measuring the optical density of the solution at 260 nm and 280 nm using NanoVue (GE Healthcare). Primer extension analysis was carried out as follows. Ten pM of the sodB_R_PriEx primer (Table 2) was labeled with ^32^P at the 5′-end by ten units of T4 polynucleotide kinase (Invitrogen) and 80 µCi of [γ-^32^P] dATP for 30 min at 37°C. The labeling mixture was heated at 70°C for 10 min and purified with MicroSpin™G-25 columns (GE Healthcare). The [γ-^32^P] end-labeled primer (0.5 pmol) was co-precipitated with 10 µg of total RNA through addition of sodium acetate and absolute ethanol. The pellet was washed with 75% ethanol, dried at room temperature, and resuspended in 20 µl of 250 mM KCl, 2 mM Tris (pH 7.9), and 0.2 mM EDTA. The mixture was heated to 65°C and then was allowed to cool to room temperature over a period of 1 hr. After annealing, 50 µl of reaction solution containing 5 µg of actinomycin D, 700 µM dNTPs, 10 mM MgCl_2_, 5 mM DTT, 20 mM Tris (pH 8.7), 30 units of RNasin (Promega), and 150 units of Superscript™ III Reverse Transcriptase (Invitrogen) was added. The mixture was incubated at 42°C for 70 min and treated with 100 U of ribonuclease T1 (Invitrogen) at 37°C for 15 min. The sample was ethanol precipitated after the addition of 1.4 µl of 5 M NaCl with 2.5 volumes of absolute ethanol and then washed with 75% ethanol. Each sample was resuspended with 6 µl of formamide dye and 4 µl of TE (pH 8.0) buffer, and then denatured at 90°C for 3 min. The samples were resolved on 6% polyacrylamide-8M urea gels, which were then dried and exposed to Imaging plate and the reverse transcription signals were analyzed using BAS 2500 (Fuji-film). The primer was also used for sequencing the *sodB* promoter region using SequiTherm EXCEL™II DNA Sequencing system (Epicentre).

### EMSA

EMSA was performed as described elsewhere with some modifications [71]. The DNA fragments containing the promoter region of *sodB*, *dps*, *luxS*, and *ahpC* were amplified by PCR with the primer pairs of SodB_F and SodB_R, Dps_F and Dps_R, LuxS_F and LuxS_R, AhpC_F and AhpC_R, respectively (Table 2). The amplified DNA was purified from an agarose gel using a gel extraction kit (Qiagen) and labeled with [γ-^32^P] ATP (GE Healthcare). The unincorporated radio isotope was removed with a MicroSpin™G-25 column (GE Healthcare). The 0.2 nM of ^32^P-labeled DNA probe was incubated with the purified rCosR protein at different concentrations (0, 0.8, 1.6, 2.4, and 3.2 nM) at 37°C for 15 min in 10 µl of the gel-shift assay buffer (20 mM HEPES (pH7.6), 1 mM EDTA, 10 mM (NH_4_)_2_SO_4_, 5 mM DTT, 0.2% Tween 20, 30 mM KCl, 0.1 µg poly (dI-dC)). For the competitive EMSA, unlabeled target DNA probes and internal cording regions of each gene were prepared by gel extraction after PCR amplification with specific primer pairs listed in Table 2. The reaction mixtures were resolved in a 6% polyacrylamide gel, and the radiolabeled DNA fragments were visualized using the BAS2500 system (Fuji Film).

### DNaseI footprinting assay

A DNaseI footprinting assay was performed following a method described previously [72]. DNA fragments containing the *sodB* and *ahpC* promoter region were amplified by PCR using a ^32^P-labeled primer (SodB_R_PriEx, AhpC_ES_R) and an unlabeled primer (SodB_F_PriEx, AhpC_FP_F) respectively (Table 2), and were purified from the agarose gel with a gel extraction kit (Qiagen). Binding of rCosR to the ^32^P-labeled *sodB* and *ahpC* promoter was performed at 37°C for 10 min in 40 µl of the gel-shift assay buffer (see above) containing 10 mM of MgCl_2_. The reaction mixture was treated either with or without 0.1 U or 0.5 U DNase I (Takara), the reactions were stopped by the addition of 200 µl of ice-cold stop solution (0.4 M NaOAc, 2.5 mM EDTA) and the DNA products were purified by phenol extraction and ethanol precipitation. The digested DNA fragments were separated by electrophoresis in 6% polyacrylamide-8 M urea gels alongside sequencing ladders that were generated with the same P^32^-labeled primer used to amplify DNA fragments for DNaseI digestion.

### Analysis of SodB enzyme activity

The SodB enzymatic activity was quantified as described elsewhere [73]. The protein samples were separated on a 12% polyacrylamide gel under native conditions. The gel was then placed into a staining solution prepared by mixing 20 ml gel staining solution A (0.05 M potassium phosphate (pH 7.8), 1 mM EDTA, 0.25 mM nitroblue tetrazolium chloride) and 1 ml gel staining solution B (0.5 mM riboflavin) in subdued light for 5 min. The gel was removed from the staining solution and placed under a fluorescent desk lamp until the gel turned uniformly purple.

### Oxidative stress sensitivity assay

To determine the sensitivity to oxidative agents, *C. jejuni* was cultured to the early-mid log phase in MH broth (approximately 5 hrs), then adjusted to an optical density of 0.2 at 600 nm (approximately 3×10^7^ cfu/ml). The aliquots of bacterial cells were exposed to the final concentration of 1 mM of paraquat and H_2_O_2_ at 42°C under microaerobic conditions for 1 hr. After exposure, cultures were serially diluted and plated on MH agar plates for viable counts.

## Supporting Information

Figure S1 **Overexpression of CosR in the** ***C. jejuni*** **strain harboring an extra copy of** ***cosR*** **in the chromosome.** The results of qRT-PCR (A) and western blotting (B) exhibited that *cosR* expression was increased in the CosR-overexpression strain. Total protein expression was visualized by SDS-PAGE as a control (lower).
(TIF)
