# Supplementary figures and images for: Regulation of Oxidative Stress Response by CosR, an Essential Response Regulator in Campylobacter jejuni
Source: PLoS One. 2011 Jul 19;6(7):e22300. doi: 10.1371/journal.pone.0022300 (PMC3139631; doi:10.1371/journal.pone.0022300)

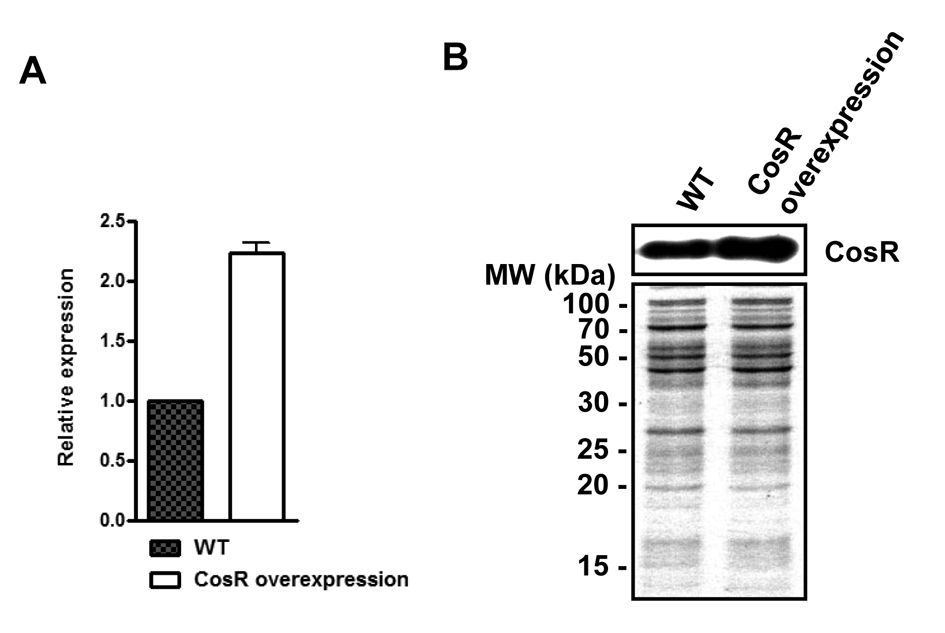

Supplement: Figure S1 — Overexpression of CosR in the C. jejuni strain harboring an extra copy of cosR in the chromosome. The results of qRT-PCR (A) and western blotting (B) exhibited that cosR expression was increased in the CosR-overexpression strain. Total protein expression was visualized by SDS-PAGE as a control (lower). (TIF) [file pone.0022300.s001.tif]
